# Reversible Causes of Transitory Motor Evoked Potential Decrease During Posterior Spine Fusion in Rapidly Progressive Scoliosis Treatment: A Case Report

**DOI:** 10.3390/diseases14030086

**Published:** 2026-02-26

**Authors:** Vladimir Djan, Vladimir Galić, Nemanja Galetić, Rastislava Krasnik, Stanislava Bodonji, Ivana Fratrić, Anna Uram Benka, Izabela Fabri Galamboš, Nikola Bošković, Jelena Mačar Novaković

**Affiliations:** 1 Faculty of Medicine, University of Novi Sad, 21000 Novi Sad, Serbiarastislava.krasnik@mf.uns.ac.rs (R.K.); ivana.fratric@mf.uns.ac.rs (I.F.);; 2 Institute of Child and Youth Health Care of Vojvodina, 21000 Novi Sad, Serbia; nemanja.galetic@izzzdiovns.rs (N.G.);; 3 Department of Neurology, University Clinical Center of Vojvodina, 21000 Novi Sad, Serbia

**Keywords:** scoliosis, spinal fusion, intraoperative neurophysiological monitoring, evoked potentials, motor

## Abstract

Introduction: Idiopathic adolescent scoliosis (IAS) is commonly managed non-surgically; however, patients with a Cobb angle >45° before skeletal maturity often require posterior spinal fusion. Because this procedure carries a risk of neurological complications, intraoperative neurophysiological monitoring (IONM) is essential for early detection of spinal cord compromise. Case report: We present a 13-year-old girl with rapidly progressing scoliosis (Cobb angle 78°) who developed intraoperative changes in motor evoked potentials (MEPs) during posterior fusion from L4 to Th2. Total intravenous anesthesia without muscle relaxants was used, and standard multimodal IONM with somatosensory evoked potentials (SSEPs), MEPs, and spontaneous/triggered electromyography was applied. After induction of general anesthesia and surgical exposure, pedicle preparation at Th8–Th9 was followed by increased bleeding from the vertebral bodies and an abrupt loss of MEPs in both lower limbs, most prominently in the tibialis anterior muscles, whilst SSEPs remained unchanged. Intraoperative radiography confirmed correct screw placement, and anesthetic variables were reassessed with no reversible cause identified. Because MEPs remained absent, a wake-up test was performed and demonstrated intact voluntary movement, allowing the surgery to continue. By the end of the procedure, MEPs recovered fully on the left side and partially on the right. The patient awoke without any postoperative motor deficit. Conclusion: It is well known that motor responses can show variability during surgery, including a gradual decrease due to prolonged anesthesia. After excluding anesthetic and mechanical factors, one of the hypothetical explanations for the transient MEP loss was temporary venous congestion and retrograde flow within the intravertebral and epidural/intraspinal venous networks, resulting in reversible spinal cord drainage impairment. Another hypothetical possibility was transient vasospasm from surgical manipulation without direct neural or vascular injury. This case highlights the critical role of continuous multimodal neuromonitoring in detecting reversible spinal cord dysfunction and guiding safe decision-making during complex scoliosis surgery.

## 1. Introduction

Managing a complex spinal deformity like idiopathic adolescent scoliosis (IAS) requires a multidisciplinary approach. In many cases, this deformity can be managed without surgery, using specialized physiatrist-prescribed exercises or the use of an orthosis to achieve correction. However, operative treatment becomes necessary for some patients. The widely accepted criterion for surgical intervention in IAS is a spinal curvature exceeding 45 degrees according to the Cobb angle before the individual reaches full skeletal maturity [1]. Regular follow-up is crucial in IAS because spinal curvature is most likely to progress during periods of rapid growth, but once skeletal maturity is reached, the deformity typically stabilizes or stops progressing altogether. When the condition worsens significantly, patients may experience functional limitations, back pain, noticeable cosmetic deformity, and a marked decline in quality of life, for which posterior spinal fusion remains the most commonly performed surgical treatment. However, it is important to recognize that this procedure carries multiple risks as it is lengthy and may involve substantial blood loss, perioperative hemodynamic instability, coagulation abnormalities, electrolyte and metabolic disturbances, and hypothermia. There is also a notable risk of temporary or permanent neurological deficits; therefore, intraoperative neurophysiological monitoring (IONM) is an essential component of such complex surgical interventions [2,3,4]. A standardized IONM protocol typically includes somatosensory evoked potentials (SEPs), motor evoked potentials (MEPs), spontaneous electromyography (sEMG), and triggered electromyography (tEMG), which is used to test pedicle screws and evaluate the integrity of the vertebral body cortex. Before neuromonitoring became the standard, Stagnara’s “wake-up test” was performed intraoperatively to assess the functional integrity of the spinal cord. Although largely replaced by modern neuromonitoring techniques, the “wake-up test” remains an indispensable method in certain cases for definitively evaluating the patient’s neurological status during surgery [5].

We present the case of a 13-year-old girl with rapidly progressing scoliosis who, during posterior spinal fusion from L4 to Th2, experienced a neuromonitoring signal drop, prompting the performance of the Stagnara “wake-up test”.

## 2. Case Report

**Figure 1:**
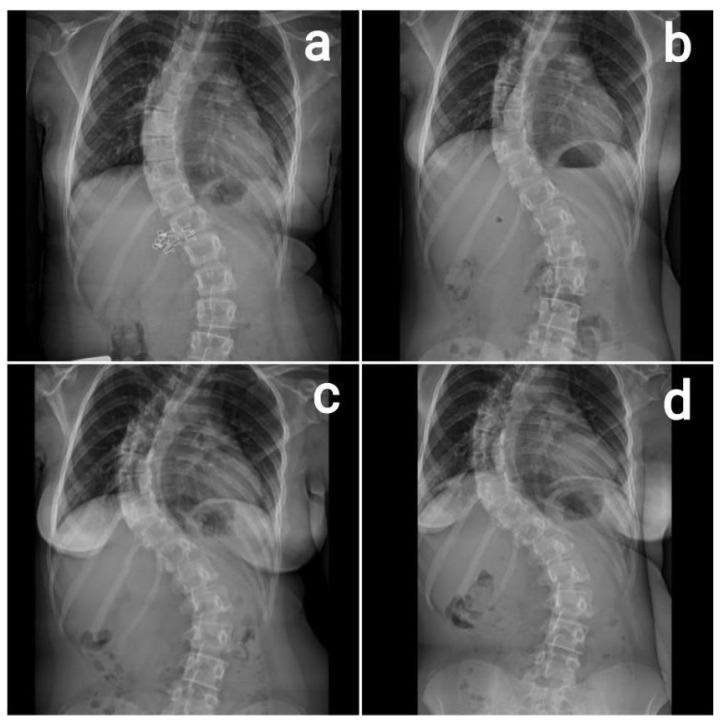
(**a**) First examination: 52 degrees by Cobb. Lenke 3C. (**b**) Three months after the first examination: 58 degrees by Cobb. Lenke 3C. (**c**) Nine months since first examination: 78 degrees by Cobb. Lenke 3C. (**d**) Last preoperative X-ray.

A 13-year-old girl presented for her initial evaluation by a pediatric orthopedist due to a spinal deformity. She had not yet reached menarche and was active in sports and piano. Clinical examination revealed left shoulder depression, asymmetry of the trunk, and a positive Adams forward-bend test. Radiographs showed a right-sided thoracic scoliosis measuring 52 degrees by Cobb, classified as Lenke type 3C (Figure 1a). She was referred to a physiatrist for corrective exercises. At a follow-up visit three months later, repeated evaluation demonstrated curve progression to 58 degrees by Cobb, still classified as Lenke 3C (Figure 1b). Menarche was still absent at that time. She continued performing the Schroth exercises prescribed by the physiatrist. Six months later, at the next follow-up visit, a further significant progression was noted, with the Cobb angle increasing to 78 degrees (Figure 1c). By this visit, menarche had occurred, and operative treatment was recommended (nine months after the initial evaluation, with a total curve progression of 26 degrees) using intraoperative neurophysiological monitoring. Prior to surgery, an additional X-ray was taken (Figure 1d).

### 2.1. Anesthesia Protocol

After appropriate preoperative preparation, the patient was reassessed on the day of surgery, her vital signs were recorded (blood pressure 100/65 mmHg, mean arterial pressure 75 mmHg, pulse 88/min, and oxygen saturation 99%), and a 22 G peripheral intravenous cannula was placed. Intravenous premedication included midazolam 3 mg, pantoprazole 40 mg, dexamethasone 4 mg, paracetamol 800 mg, and tranexamic acid 1000 mg. The patient was subsequently transferred to the operating room, where standard monitoring (electrocardiography (ECG), noninvasive blood pressure, and pulse oximetry) was initiated. Intravenous induction was achieved with propofol at 2.7 mg/kg, fentanyl at 1.8 mcg/kg, and rocuronium at 0.54 mg/kg. After endotracheal intubation, two additional peripheral intravenous (IV) lines (20 G and 18 G) were placed, along with an arterial line for invasive blood pressure monitoring, a nasogastric tube, a urinary catheter, and a temperature probe. Active warming was continuously applied using a forced-air warming system, and intravenous fluids were warmed to maintain normothermia and minimize temperature-related variability in intraoperative neuromonitoring. Bispectral index (BIS) electrodes were applied for anesthesia depth monitoring, and electrodes for intraoperative neuromonitoring were also positioned accordingly. Anesthesia was maintained with sevoflurane (minimum alveolar concentration (MAC) 0.6–0.7 Vol%) and FiO_2_ 0.5 until the patient was placed in the prone position. After repositioning, the inhalational agent was discontinued, and total intravenous anesthesia (TIVA) was initiated using a continuous infusion of propofol at 116.6 mcg/kg/min and remifentanil at 0.5 mcg/kg/min. During the remainder of the procedure, no additional muscle relaxants were administered. BIS values ranged from 38 to 46. To minimize the risk of massive bleeding, a continuous infusion of tranexamic acid at 2 mg/kg/h was maintained, along with controlled hypotension using a continuous infusion of sodium nitroprusside at 0.2–0.5 mcg/kg/min, targeting a mean arterial pressure (MAP) of 55–65 mmHg.

At the 160th minute of surgery, the neurologist reported a loss of motor evoked potentials in the left leg. At that time, the patient was hemodynamically stable, with blood pressure at 90/50 mmHg (MAP 63), pulse at 72/min, oxygen saturation at 100%, a BIS of 42, and a body temperature of 35.5 °C. Recent laboratory and arterial blood gas analyses showed no significant abnormalities, with hematocrit 0.30, hemoglobin 101 g/L, and a base excess of −5.4 (BE_ecf), while pH remained within the reference range. The sodium nitroprusside infusion was then discontinued, resulting in a spontaneous rise in blood pressure to 100/55 mmHg (MAP 70) within a few minutes.

### 2.2. IONM Protocol

An ISIS system (Inomed Co., Emmendingen, Germany) was used for stimulation and recording. SEPs were evoked by stimulation of the medial and tibial nerves, at the level of the wrist and ankle joint, respectively (single impulse, duration 0.2 ms, frequency 4.3 Hz, intensity 20, respectively 30 mA), and recording was performed using spiral electrodes placed on the patient’s scalp at positions CzFz and C3/C4-Fz, in accordance with the international 10–20 system. MEPs were obtained by transcranial electrical stimulation with a multiphase technique and a short series of six pulses (with a single-pulse duration of 0.5 ms, an interstimulus interval of 4 ms, and a frequency of 2 Hz), via spiral electrodes placed above C3/C4 and using a constant current stimulator with a maximum output of up to 200 mA. MEPs were recorded using needle electrodes placed at a distance of 1 cm in the muscles of the upper (abductor pollicis brevis) and lower extremities (tibialis anterior [L4-5], vastus medialis [L3-4], adductor magnus [L2-3]), as well as in m. rectus abdominis [Th12-8] and intercostal muscles [Th7-4] bilaterally. The same electrodes were used to record sEMG activity and tEMG responses from muscle groups receiving innervation from nerve roots at risk of injury during the placement of pedicle screws. Testing of the pedicle screws was performed with single-pulse constant current stimulations (with a pulse duration of 0.2 ms and a frequency of 1 Hz) with variable intensities. The intensity was gradually increased from 0 mA in steps of 2–3 mA until no evoked responses were recorded or a maximum intensity of 30 mA was reached. A stimulation threshold of 8 mA was taken as a neurophysiological marker of a properly placed pedicle screw and an intact pedicle cortex. The baseline sensitivity for signal acquisition was set to 50 μV per division. After proper positioning of the patient, and before the initial skin incision, basal SEP and MEP values were recorded according to the standard IONM protocol. During opening and initiation of spinal instrumentation placement, SEP and MEP were routinely checked every 5–7 min and subsequently after the placement of each pedicle screw. During and after the placement of each screw, sEMG and tEMG were used to detect any unusual events. During the critical phases of scoliosis correction, MEPs were monitored regularly every 2–3 min and this continued for 30 min following the completion of the spinal correction.

### 2.3. Operating Procedure

**Figure 2:**
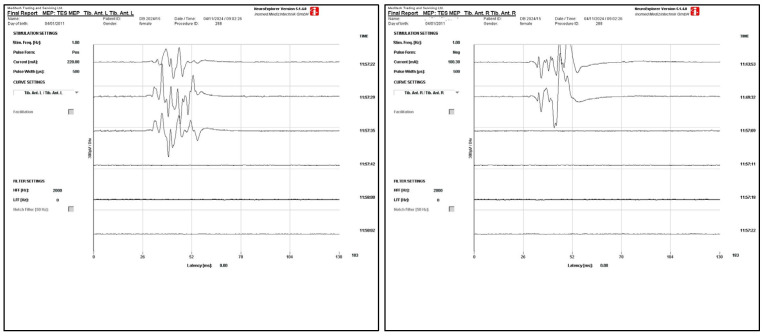
Loss of MEP responses—Tib. anterior left and right.

After the induction of general anesthesia, the patient was positioned prone with soft padding applied at areas of highest pressure. Following standard preparation of the surgical field, a midline incision was made from Th2 to L4, and the paravertebral musculature was dissected, although the vertebrae were not yet fully exposed. Bilateral transpedicular screws were inserted from L4 to Th5, but not Th8, where a screw was placed only on the left side. Pedicle hooks were placed bilaterally at Th3, and laminar hooks were placed bilaterally at Th2. During pedicle preparation for screw placement at Th8 and Th9, slightly increased bleeding from the vertebral bodies was noted. Immediately after screw placement at these levels, there was a sudden loss of MEP responses in both lower extremities (Figure 2), most prominently in the tibialis anterior myotome, while SEP parameters remained unchanged.

**Figure 3:**
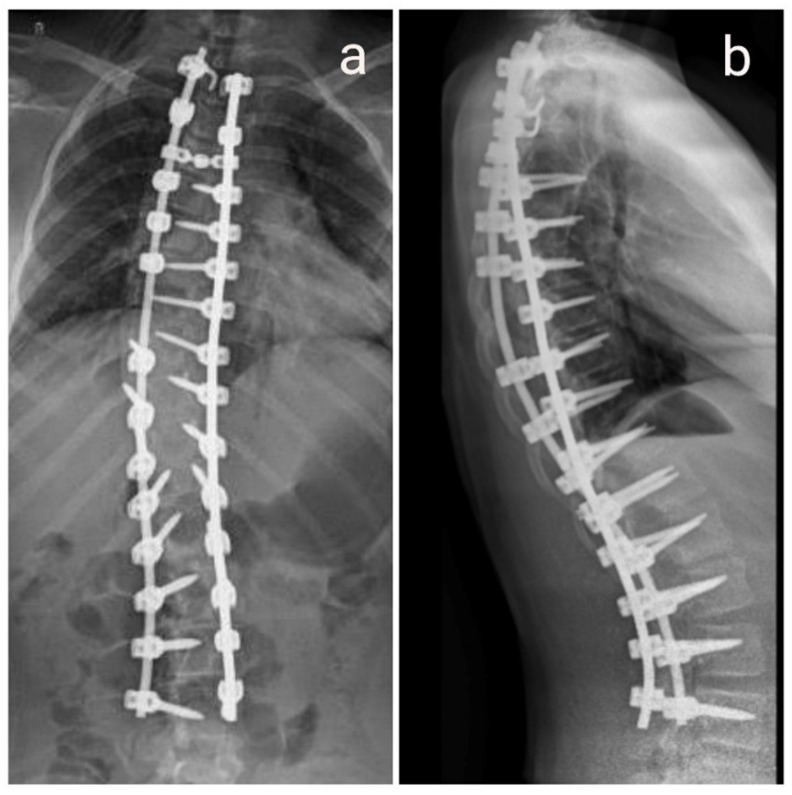
Postoperative X-ray. (**a**) AP X-ray image. (**b**) L X-ray image.

An intraoperative audit of the screws and X-ray imaging confirmed that all implants were correctly positioned. Anesthetic parameters were thoroughly reviewed to rule out any factors that could interfere with IONM recordings. All the electrodes were replaced and the proper functioning of the neuromonitoring device was checked. Since MEP responses from the lower extremities remained absent a decision was made to perform a “wake-up test.” Propofol infusion was discontinued, and the remifentanil infusion was reduced to 0.2 mcg/kg/min. It took twenty minutes from stopping propofol until the patient reached an adequate level of consciousness and understood verbal commands appropriately. At that time, her blood pressure was 110/65 mmHg (MAP 80), pulse was 80/min, oxygen saturation was 100%, and BIS was 78. After confirming a satisfactory motor response with voluntary dorsiflexion of both feet, a 100 mg bolus of propofol was administered, and a continuous propofol infusion was resumed at the pre-test rate, while the remifentanil infusion was increased back to its previous dose. During the remainder of the procedure, the patient remained hemodynamically stable without the need for sodium nitroprusside, with MAP maintained at around 65 mmHg. The surgery proceeded with placement of the remaining transpedicular screws from Th7 to Th4, along with pedicle and laminar hooks, followed by insertion of bilateral cobalt chromium rods (Co-Cr), achieving scoliosis correction. The surgical wound was closed in anatomical layers, with subcutaneous tissue sutured using continuous Vicryl 4-0 and the skin closed with interrupted Ethilon 3-0 sutures. By the end of the operation, MEP responses in the left lower limb had fully recovered to baseline values, while responses in the right lower limb partially returned to their initial levels. After surgery, the patient was awakened and extubated on the operating table without any motor neurological deficits, and she was admitted to the Department of Surgical Intensive Care. On the first postoperative day, she was transferred to the Department of Orthopedics and assisted to sit on the side of the bed. By the second postoperative day, she was able to stand beside the bed. On the same day, a consultant neurologist evaluated her and confirmed normal neurological function. The urinary catheter was removed on the third postoperative day. A follow-up X-ray was performed on the eighth postoperative day (Figure 3), after which the patient was discharged.

## 3. Discussion

It has been scientifically established that sex hormones can contribute to the progression of spinal curvature in children with idiopathic scoliosis (IS). In girls, the timing of menarche is particularly relevant. A study of 208 patients, in which the mean age at menarche was 154.8 ± 14.7 months, found that girls with more severe forms of IS tended to experience menarche later than those with milder forms [6]. In our patient, over a period of nine months from the initial evaluation (at 156 months), the Cobb angle progressed by 26 degrees, indicating rapid clinical deterioration prior to menarche (which occurred at 168 months), which aligns with reports from the literature. Consequently, the decision to proceed with surgical intervention should ideally be made after menarche and once the period of rapid curve progression has stabilized.

At our institution, intravenous midazolam is routinely administered prior to induction of general anesthesia; however, it is known to reduce MEP amplitudes by up to 20% [7]. The advantage of midazolam is its relatively short half-life of approximately two hours, which allows for its effects to subside before the placement of transpedicular screws, the critical phase of the procedure [7]. Considering the well-documented impact of inhalation anesthetics on evoked potentials, anesthesia was initially maintained with an inhalation agent until the patient was positioned prone, after which total intravenous anesthesia (TIVA) was initiated to ensure optimal intraoperative neuromonitoring in line with standardized protocols. Muscle relaxants were used only for intubation, consistent with recommendations in the literature [4,8,9]. Throughout the procedure, hemodynamic stability was well maintained, with MAP values ranging from 55 to 65 mmHg, consistent with recommendations in the literature [10,11]. At the time of MEP loss, the patient remained hemodynamically stable, and mild hypothermia (35.5 °C) was not considered sufficient to interfere with neuromonitoring. Hemoglobin and hematocrit levels were also adequate for reliable IONM recording. After thoroughly checking and optimizing all factors that could potentially affect neuromonitoring, the team elected to perform the wake-up test. In current practice, optimization of hemodynamics, reversal of corrective maneuvers, implant reassessment, and technical troubleshooting are typically performed before or alongside the wake-up test, which is the method recommended in the literature for situations of persistent MEP loss [12,13]. The wake-up test presents a significant challenge for the anesthesiologist, primarily due to the patient being in a prone position, which increases the risk of accidental extubation and air embolism, as well as the need to rapidly achieve a sufficient level of consciousness to perform the test while maintaining adequate analgesia [12,14]. In our case, anesthesia was maintained with a combination of propofol infusion and the short-acting opioid remifentanil, consistent with most reported protocols. This approach allowed the wake-up test to be performed quickly, safely, and with effective analgesia [15,16]. As part of standard monitoring, BIS was used to assess the depth of anesthesia. The patient responded to verbal commands with a BIS value of 78 after twenty minutes, which is notably shorter than the awakening time reported by Madhavi et al., where balanced anesthesia was used and the mean awakening time was 42.60 ± 12.51 min [17]. Although Rehberg et al. reported that the use of remifentanil infusion during scoliosis surgery with a wake-up test may be associated with a higher incidence of postoperative sleep disturbances, no such complications were observed in our patient [18]. BIS monitoring facilitated controlled emergence during the wake-up test by providing an objective estimate of anesthetic depth. Compared with other modalities, such as entropy or end-tidal anesthetic concentration, BIS offers rapid feedback and, when combined with clinical assessment, supports safe and timely awakening.

In cases of persistent MEP loss despite optimization of hemodynamics, verification of instrumentation, and exclusion of technical causes, a wake-up test remains an important confirmatory method recommended in selected situations [19].

It is well known that MEP responses can show variability during surgery including a gradual weakening of the MEP signal due to prolonged anesthesia, the exact mechanism of which is not well known [20]. Although we took this into consideration, this cause of motor evoked potential loss was less likely in our case. During pedicle preparation for screw placement, slightly increased bleeding was observed from the vertebral bodies of the eighth and ninth thoracic vertebrae, which could suggest the presence of a hemangioma in the differential diagnosis. However, since no preoperative MRI was performed, the exact cause of the bleeding could not be determined, representing a limitation of this case. In similar future cases, MRI assessment should be included for preoperative imaging in rapidly progressive idiopathic scoliosis for detection of possible spinal hemangiomas or other vascular anomalies.

Primary spinal tumors are rare, often asymptomatic, and their true incidence is not well established. Hemangiomas and enostoses are considered the most common primary spinal tumors, with an estimated incidence of 11–14% [21], and radiological imaging plays a crucial role in diagnosing vertebral hemangiomas. While anteroposterior X-rays can sometimes reveal these lesions as coarse vertical striations, this was not observed in our patient’s X-rays. Moreover, for detection via this method, a hemangioma typically needs to occupy at least one-third of the vertebral body [22].

After excluding all possible causes from the aspect of anesthesia and mechanical causes related to the insertion of pedicle screws, the hypothetical explanation of the intraoperative loss of MEP could be a transient venous stasis and possible retrograde blood flow between the intravertebral and extradural/intraspinal venous networks. This process could have caused congestion of the spinal cord, obstruction of the venous drainage flow, and consequent transient loss of the MEP response. Apart from the above, transient and localized vasospasm due to harmless surgical manipulation and without direct trauma to the blood vessel, dura, or spinal cord can consequently lead to localized hypoperfusion, which may cause transient loss of motor evoked potentials. However, as no direct perfusion assessment nor MRI imaging confirmation was performed in our case, we cannot state with certainty that these were probable causes of loss of MEP response; therefore, this explanation remains hypothetical. Additional research on this subject should add more clarification about potential pathophysiological mechanisms in cases like this. MEP usually recovers when timely steps are taken immediately [23]; however, the extent to which the spinal cord can tolerate decreased levels of perfusion pressure is unpredictable and still a topic of ongoing scientific debate. This case report has several limitations. Preoperative MRI was not performed, which limited the ability to exclude vertebral hemangioma or other vascular anomalies. In addition, no direct intraoperative perfusion monitoring was available to confirm the proposed pathophysiological mechanism. Finally, as a single-case report, the findings cannot be generalized but may serve to highlight potentially reversible causes of intraoperative MEP loss.

## 4. Conclusions

Our case suggests that in susceptible patients a transient loss of MEP can occur independently of surgical manipulation or anesthesia intervention due to hypothetical and transient events, such as venous stasis and/or localized transient vasospasm. Early recognition of such events is crucial, as is the sequence of actions needed to identify the true cause of MEP loss and to make timely, expert decisions in the best interests of the patient.

## Data Availability

The data presented in this study is available on request from the corresponding author.

## Abbreviations

IJS	idiopathic juvenile scoliosis
IONM	intraoperative neurophysiological monitoring
MEP	motor evoked potentials
SSEP	somatosensory evoked potentials
sEMG	spontaneous electromyography
tEMG	triggered electromyography
ECG	electrocardiography
IV	intravenous
TIVA	total intravenous anesthesia
BIS	bispectral index
MAC	mean alveolar concentration
MAP	mean arterial pressure
